# Reconfigurable fiber-to-waveguide coupling module enabled by phase-change material incorporated switchable directional couplers

**DOI:** 10.1038/s41598-022-11386-3

**Published:** 2022-05-04

**Authors:** Bishal Bhandari, Sang-Shin Lee

**Affiliations:** Department of Electronic Engineering, Kwangwoon University, Seoul, 01897 South Korea

**Keywords:** Optics and photonics, Integrated optics

## Abstract

In silicon photonics, grating-assisted fiber-to-waveguide couplers provide out-of-plane coupling to facilitate wafer-level testing; however, their limited bandwidth and efficiency restrict their use in broadband applications. Alternatively, end-fire couplers overcome these constraints but require a dicing process prior usage, which makes them unsuitable for wafer-level testing. To address this trade-off, a reconfigurable fiber-to-waveguide coupling module is proposed and designed to allow for both grating-assisted and end-fire coupling in the same photonic circuit. The proposed module deploys a switchable directional coupler incorporating a thin layer of phase-change material, whose state is initially amorphous to render the coupler activated and hence facilitate grating-assisted coupling for wafer-level testing. The state can be altered into crystalline through a low-temperature annealing process to deactivate the directional coupler, thus facilitating broadband chip-level coupling through end-fire couplers. All the components encompassing conjoined switchable directional couplers as well as the grating and end-fire couplers were individually designed through rigorous simulations. They were subsequently assembled to establish the proposed reconfigurable coupling module, which was simulated and analyzed to validate the selective coupling operation. The proposed module gives rise to a low excess loss below 1.2 dB and a high extinction ratio over 13 dB throughout the C-band, when operating either under grating-assisted or end-fire input. The proposed reconfigurable coupling module is anticipated to be a practical solution for flexibly expediting the inspection of integrated photonic circuits on a wafer scale.

## Introduction

Photonic integrated circuits (PICs) for quantum computing, beam steering, optical communications, and many other applications have been extensively researched and developed over the recent decade^1–6^. From the perspective of practical inspection and operation of the photonic circuits, a grating-assisted coupler (GAC) and an end-fire coupler based on a spot size converter (SSC) are mainly regarded as the popularly recommended schemes for fiber-to-waveguide light coupling^7–12^. The GACs are primarily geared for out-of-plane coupling, whereas the SSCs are suitable for in-plane end-fire coupling^7,8^. Wafer-level testing, which is only possible with the help of the GACs, is essential to ensuring the best PIC yield during fabrication. However, for the GACs, the limited bandwidth, lower coupling efficiency, and higher sensitivity to polarization render them less desirable^7,9^. On the other hand, the SSCs give rise to an outstanding coupling efficiency, broader bandwidth, and better polarization tolerance^7,13^, but inevitably entail an additional process of dicing/polishing the chips; thus making them unsuitable for wafer-level testing. With the burgeoning demand on highly integrated photonic chips and the rapidly increasing size of photonic wafers, wafer-level testing has become understandably indispensable for expediting the chip development process. In this context, the choice between the GAC and SSC incites a trade-off between the fabrication quality and PIC usability. Therefore, a method that incorporates both couplers into the same circuit would be highly beneficial for achieving better fabrication quality as well as wider applicability.

Over the past few decades, several studies on end-fire and grating couplers have been conducted separately; however, to the best of our knowledge, there was no reported work discussing an integrated coupler that can cater to both in-plane coupling resorting to an end-fire coupler and out-of-plane coupling utilizing a GAC in the same circuit. To integrate the GAC and SSC into the same circuit simultaneously, a mechanism that switches the inputs and outputs is necessary. It is unfeasible to use the commonly available photonic switches relying on thermo-optic and electro-optic effects, since their operation entails a continuous supply of power for their operation^3^. With the advent of optical phase-change materials (PCMs), nonvolatile switching can be executed with no continuous power supply^14–18^. The adoption of PCMs makes it possible to simultaneously integrate GAC and SSC into a single circuit, thereby rendering both wafer-level and chip-level coupling of light. A study reported by Y. Zhang group demonstrated a transient tap coupler capable of tapping a small portion of light from a waveguide, which could be disabled by adjusting the state of the PCM, for probable applications in wafer-level testing^19^. However, it is limited to tapping only a small portion of light, not facilitating full-scale wafer level testing of circuits. Full-scale characterization, which can deal with an input/output power rather than a small portion thereof, is integral to taking full advantage of wafer-level testing. In this context, a reconfigurable coupling scheme that provides for both full-scale wafer-level and chip-scale testing is categorically sought for to execute speedy and flexible inspection of PICs under production.

In this paper, we propose and design a reconfigurable fiber-to-waveguide coupling (RFWC) module based on a PCM overlaid switchable directional coupler (SDC), which serves both in-plane coupling mediated by an SSC and out-of-plane coupling relying on a GAC in the same photonic circuit. A pair of PCM-incorporated SDCs is deployed to route the light from these two disparate couplers into and out of the chip. The state of the PCM can be switched between amorphous and crystalline states and vice versa, by applying heat or high-energy light pulses to the chip at a specific temperature under a specific environment^6,15,19,20^. This invokes nonvolatile switching when used with directional couplers. Because the state of the PCM remains in its current state unless its state is altered by the application of heat or high-energy light pulses^6,20^, the directional coupler requires no continuous heat/power supply for operation. The proposed coupling module guides the input/output through the GACs, facilitating full-scale wafer-level testing until the SDC is switched into input/output coupling via the SSCs, thereby achieving chip-level interrogation. The proposed RFWC module is categorically anticipated to usher in various types of advanced flexible coupling schemes for inspecting PICs by accommodating different types of interrogation schemes.

## Proposed reconfigurable fiber-to-waveguide coupling module and its design

**Figure 1:**
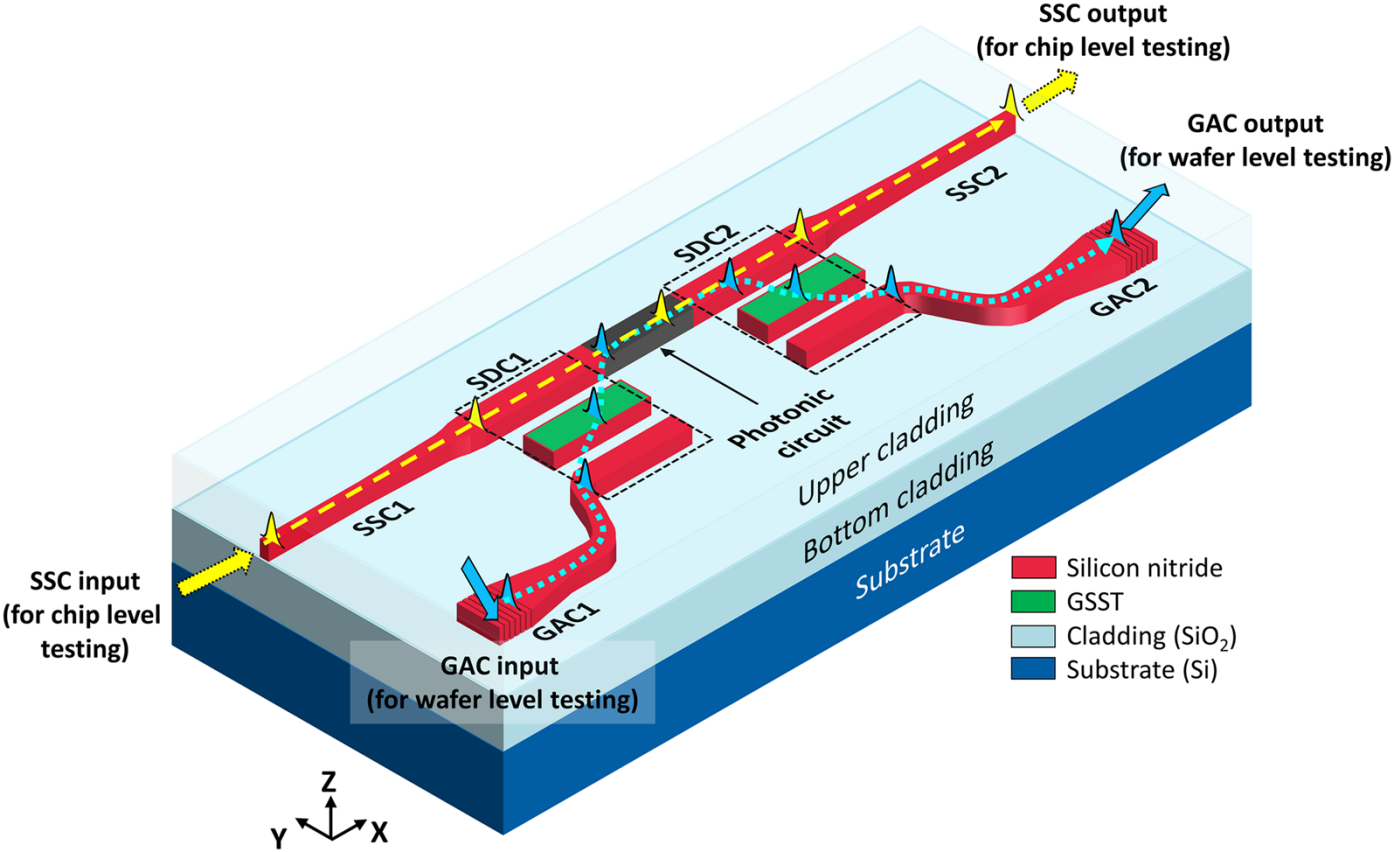
Illustration of the proposed RFWC module. Light fed from the input SSC1 propagates through the photonic circuit and exits through the output SSC2 (indicated by yellow line) when both SDC1 and SDC2 are deactivated. When they are activated, SDC1 and SDC2 couple the light into and out of the photonic circuit through the input and output GACs (marked by blue dotted lines).

An illustration of the proposed RFWC module is shown in Fig. 1. It consists of a pair of PCM-overlaid switchable directional couplers (SDC1 and SDC2) connected to a pair of GACs (GAC1 and GAC2) and SSCs (SSC1 and SSC2) at both input and output facets. The two SDCs are interfaced at either end of the photonic circuit to facilitate selective switching between the GAC and SSC inputs/outputs. For the proposed RFWC module, a straight waveguide was considered as the photonic circuit under investigation, which may be replaced by other photonic circuits in future applications. Both the SDCs exploits a thin layer of PCM, whose state can be altered from amorphous to crystalline and vice versa by virtue of low-temperature annealing in the vicinity of 280 °C^15,19^. The SDC is activated when the PCM is amorphous and deactivated when it is crystalline. This SDC switching permits the input and output from either the GAC or SSC to be activated and deactivated. In this work, Ge_2_Sb_2_Se_4_Te_1_ (GSST) was chosen as the PCM candidate owing to its lower optical loss in both the amorphous and crystalline states compared to other PCMs^15,19^. At a wavelength (λ) of 1550 nm, the amorphous state of GSST (aGSST) exhibits a refractive index of 3.3258 in conjunction with a small extinction coefficient of 1.8 × 10^−4^ while its crystalline state (cGSST) manifests a higher refractive index and extinction coefficient of 5.083 and 0.35, respectively^21^. On account of the high index contrast between the amorphous and crystalline states of the PCM, a substantially high on–off ratio and wide tunability can be achieved^17^. Since the GSST remains in the amorphous state in its original form, the SDC is designed to operate under the combination of GAC1 input and GAC2 output by default, which can facilitate wafer-level testing. In the amorphous state, the transverse electric (TE) polarized input from GAC1 undergoes cross-coupling via SDC1 into the photonic circuit and couples back to the output GAC2 via SDC2. In the meantime, the RFWC module could be reconfigured for end-fire coupling by changing the state of the GSST into the crystalline state. In this case, the TE input from SSC1 can enter the photonic circuit and exit the SSC2 output without going through cross-coupling at the SDCs. The design and performance of components constituting the proposed RFWC module, including the SDC, GAC, and SSC, are individually discussed in the following sections. Finally, the proposed complete RFWC module has been built and meticulously evaluated.

### Design of the switchable directional coupler

**Figure 2:**
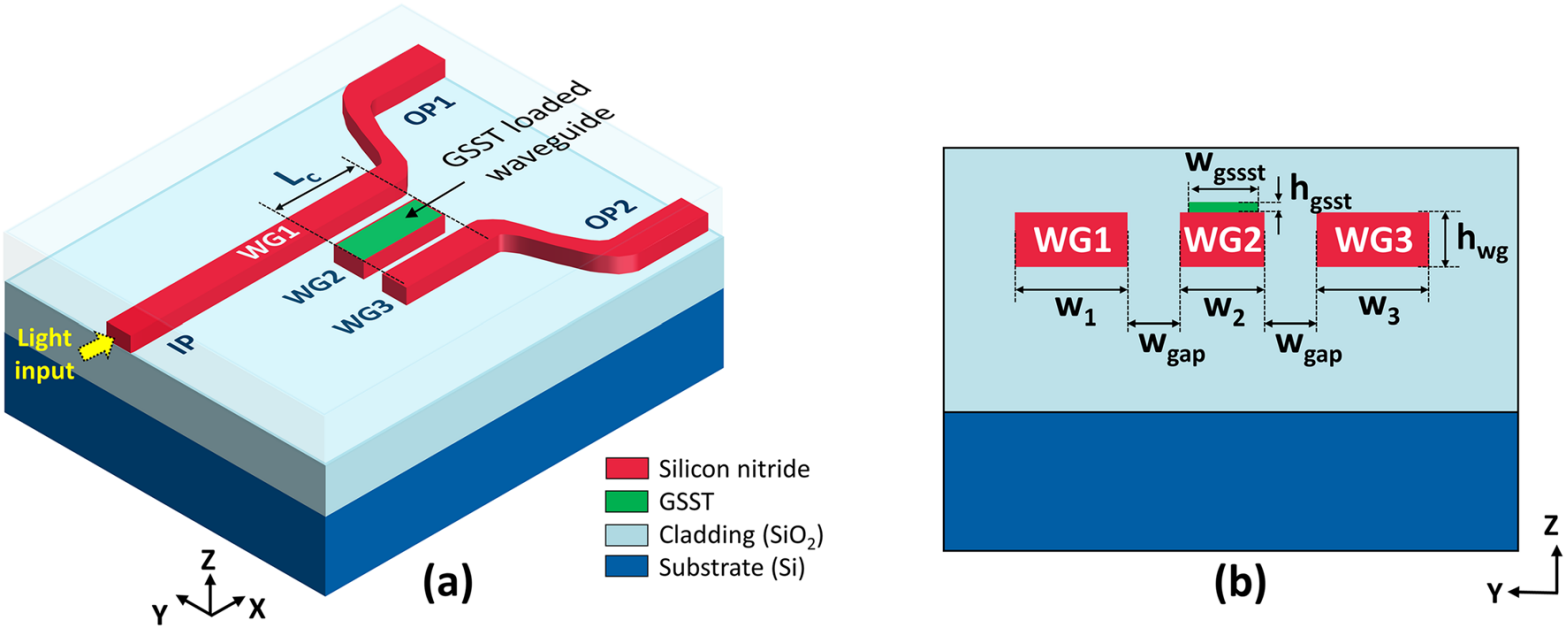
(**a**) Proposed SDC incorporating two identical waveguides WG1 and WG3, which are mediated by a GSST loaded waveguide WG2 in the coupling region of a length L_c_. (**b**) Cross-sectional view of the SDC with the structural parameters indicated.

Directional couplers play an integral role in the proposed RFWC module, considering they allow light from different input couplers to enter the circuit and subsequently exit through their respective output couplers. Toward that end, a three-waveguide SDC was concocted based on a silicon nitride (SiN, n =  ~ 2.0) waveguide platform capitalizing on a silicon dioxide (SiO_2_, n = 1.44) cladding. Figure 2a shows the proposed SDC structure which comprises two identical SiN strip waveguides (WG1 and WG3) mediated by a GSST-loaded WG2 in the coupling region, forming a three-waveguide directional coupler. The waveguide WG2 has a length of L_c_, which stands for the coupling length required to achieve complete power transfer from WG1 to WG3 through the GSST-loaded WG2 under the aGSST state. The outputs from WG1 and WG3 relate to output ports OP1 and OP2, respectively. The cross section of the directional coupler is depicted in Fig. 2b. The widths of waveguides WG1, WG2, and WG3 were w_1_, w_2_, and w_3_, respectively. w_gsst_ is the width of the GSST layer placed atop the WG2 waveguide, which is 20 nm narrower than w_2_, in consideration of practical alignment tolerance during fabrication. The thicknesses of the SiN waveguide (h_wg_) and GSST layer (h_gsst_) were determined to be 500 and 50 nm, respectively. The gap (w_gap_) between the waveguides were set to be identical.

**Figure 3:**
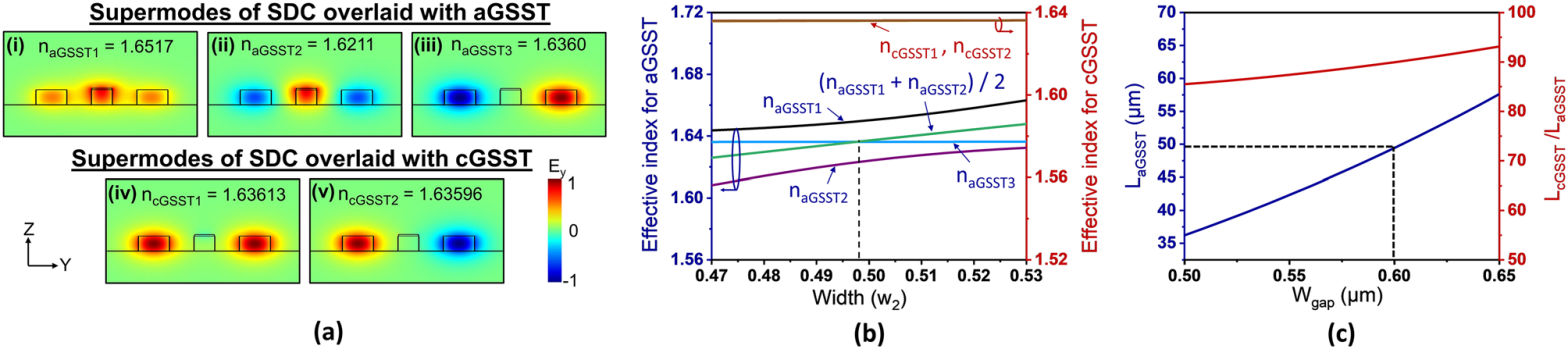
(**a**) Calculated supermode profiles of the SDCs overlaid with aGSST (i)–(iii) and cGSST (iv)–(v). (**b**) Corresponding effective refractive indices of the supermodes in terms of the width w_2_. (**c**) Calculated coupling length L_aGSST_ and ratio of L_cGSST_ to L_aGSST_ with respect to the gap (w_gap_).

The optimal parameters of the widths and gap of the SDC were determined by meticulously analyzing the effective refractive indices of its eigenmodes. To ensure single-mode operation for a 500-nm thick SiN waveguide^5^, the w_1_ and w_3_ values were identically set at 750 nm. Figure 3a portrays the TE field distribution of the supermodes pertaining to the SDC at λ = 1550 nm for both the aGSST and cGSST states. It was observed that the aGSST-loaded SDC supported two symmetric modes and one asymmetric mode corresponding to effective refractive indices of n_aGSST1_, n_aGSST2_, and n_aGSST3_, respectively, as shown in Fig. 3a-(i)–(iii). The cGSST-overlaid SDC could guide a symmetric and asymmetric supermode with effective indices of n_cGSST1_ and n_cGSST2_, respectively, as shown in Fig. 3a-(iv) and (v). The effective indices of the supermodes for different w_2_ values are shown in Fig. 3b. The maximum power transfer from WG1 to WG3 transpires when the effective indices of the supermodes satisfy the phase-matching condition, which is given by ${\text{n}}_{{{\text{aGSST1}}}} + {\text{n}}_{{{\text{aGSST2}}}} = {\text{2n}}_{{{\text{aGSST3}}}}$^22,23^ and satisfied when w_2_ = 497 nm for the aGSST-loaded SDC. For the case of cGSST-loaded SDC, however, the effective indices were invariant to w_2_, leading to no phase-matching condition. The coupling characteristics with respect to the gap width (w_gap_) was then scrutinized. The coupling lengths leading to complete power transfer between WG1 and WG3 for SDCs loaded with aGSST and cGSST are denoted by L_aGSST_ and L_cGSST_, respectively. The coupling lengths, which are estimated from ${\text{L}}_{{{\text{aGSST}}}} { = }\lambda {\text{/2(n}}_{{{\text{aGSST1}}}} {\text{ - n}}_{{{\text{aGSST3}}}} {)}$ and ${\text{L}}_{{{\text{cGSST}}}} { = }\lambda {\text{/2(n}}_{{{\text{cGSST1}}}} {\text{ - n}}_{{{\text{cGSST2}}}} {)}$^16^, were calculated at λ = 1550 nm; the values of L_aGSST_ and the ratio of L_cGSST_ to L_aGSST_ are plotted in Fig. 3c. Both L_aGSST_ and L_cGSST_/L_aGSST_ values increased with the gap. A higher value of L_cGSST_/L_aGSST_ translates into a higher extinction ratio and is achieved at a wider gap; however, a larger gap increases the L_aGSST_, implying a larger device footprint. From the perspective of the footprint and performance trade-off, a gap of 600 nm was selected for the proposed coupler. Consequently, the coupling length was determined to be L_c_ = L_aGSST_ = 49.5 µm, leading to an optimal power transfer between WG1 and WG3 under aGSST state. The achieved coupling length is short enough from the perspective of typical photonic circuits; the ratio of L_cGSST_/L_aGSST_ was found to be approximately 91, which is appropriate to facilitate the optical switching leading to a high extinction ratio.

**Figure 4:**
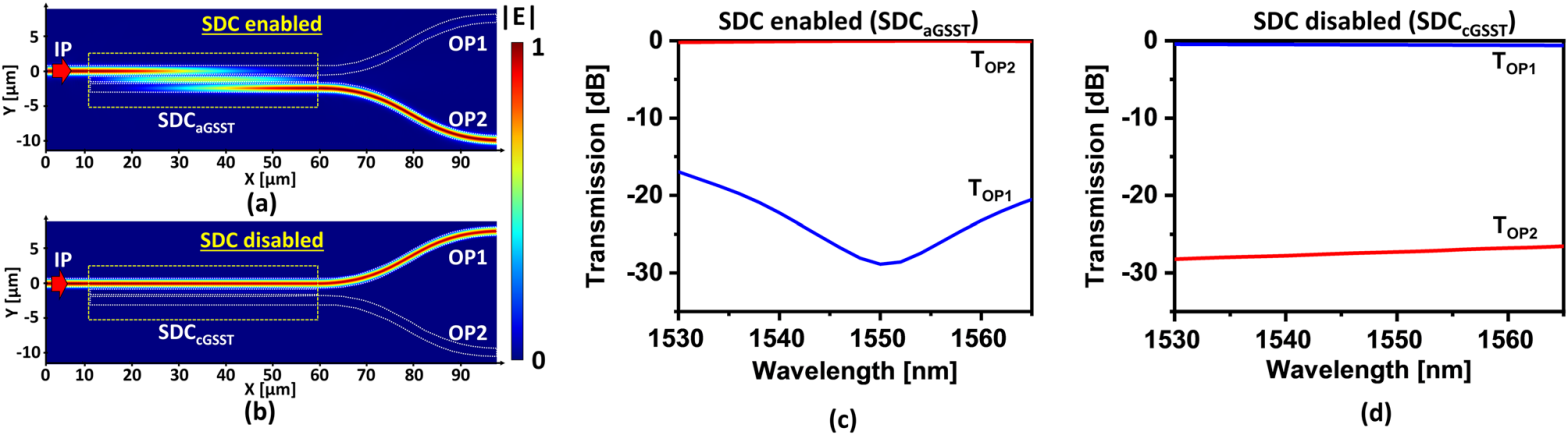
(**a**) Coupling of light from the input port IP to the output port OP2 when the SDC is operated under aGSST state. (**b**) Under cGSST state, no coupling occurs, and the output exits OP1. The spectral transmission of the two ports is observed for the SDC-enabled and disabled cases of (**c**) aGSST and (**d**) cGSST, respectively.

Next, using the designed structural parameters, the SDC was modeled and simulated with the aid of a 3D finite-difference time-domain (FDTD) simulation tool. Figures 4a,b sketch the light propagation through the SDC when it is enabled and disabled, respectively. The SDC is enabled when it is operated under aGSST (denoted by SDC_aGSST_). It lets incident light from the input port (IP) be cross coupled through the SDC and exit through output port OP2. Meanwhile, for the SDC operating under cGSST (denoted by SDC_cGSST_), the input light IP undergoes no cross-coupling due to lack of phase matching, hence exiting port OP1. The performance of a directional coupler is usually characterized in terms of insertion loss (IL) and extinction ratio (ER). The IL signifies the amount of loss incurred between the input port and the targeted output port, while the ER is the ratio of the output powers transmitted through the two output ports. The IL and ER can be estimated in accordance with ${\text{IL}}_{{\text{aGSST(cGSST)}}} {\text{ = 10 log}}_{{{10}}} {\text{T}}_{{\text{OP2(OP1)}}}$ and ${\text{ER}}_{{\text{aGSST(cGSST)}}} {\text{ = 10 log}}_{{{10}}} {\text{T}}_{{\text{OP2(OP1)}}} {\text{/ T}}_{{\text{OP1(OP2)}}}$, respectively. The transmission spectral response of the SDC_aGSST_ is shown in Fig. 4c. The SDC_aGSST_ was seen to attain an IL_aGSST_ of as low as 0.04 dB and an ER_aGSST_ of as high as 28.8 dB at λ = 1550 nm. For the operation under the C-band spectrum (λ = 1530 nm–1565 nm), an IL_aGSST_ of below 0.3 dB and an ER_aGSST_ of above 17 dB were obtained. Also, the transmission spectral response of the SDC_cGSST_ is shown in Fig. 4d. The IL_cGSST_ and ER_cGSST_ were 0.5 and 27 dB at λ = 1550 nm, respectively. The IL_cGSST_ and ER_cGSST_ were witnessed to stably remain below 0.6 dB and beyond 25 dB throughout the C-band, respectively. It is noteworthy that the device performance in terms of the IL and the ER are squarely susceptible to refractive index variations in GSST in action. Hence, the switching of states of GSST should be meticulously executed under specific conditions^19,21^, thereby efficiently preventing undesired refractive index variations incurred by incomplete state-switching.

### Design of the grating-assisted coupler and the end-fire coupler

**Figure 5:**
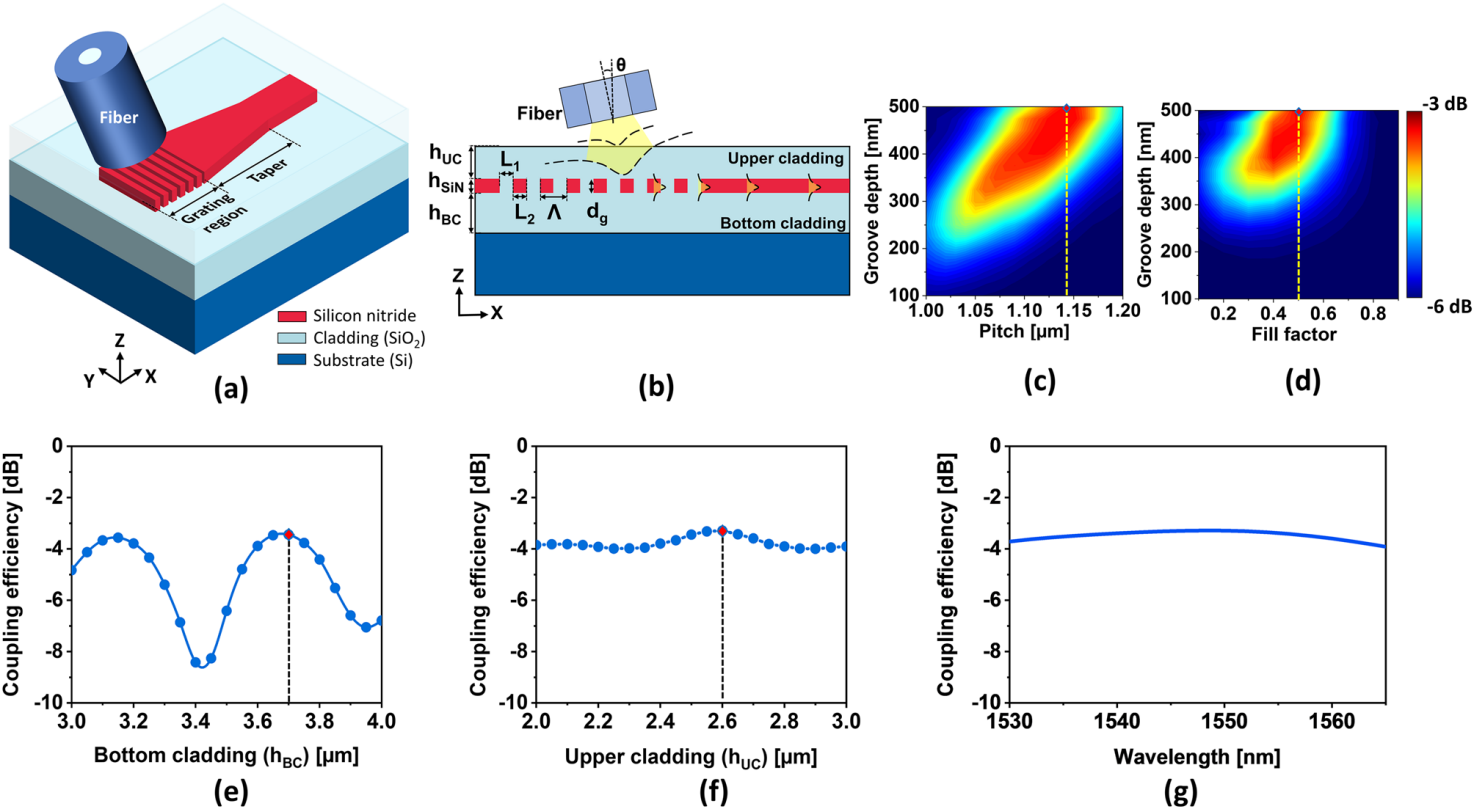
(**a**) Schematic of the proposed GAC and (**b**) its cross-sectional view with the structural parameters labeled. (**c**) Calculated coupling efficiency with respect to the pitch and groove depth. (**d**) Coupling efficiency depending on the fill factor and groove depth. Coupling efficiency as function of (**e**) the bottom and (**f**) upper cladding. (**g**) Spectral coupling response under the specified design parameters.

As a proof of concept, a GAC and an SSC for end-fire coupling were attempted for the simulations of the proposed RFWC module. As shown in Fig. 5a, the GAC is comprised of a grating region and taper to convey incident light toward a 750-nm wide input waveguide. In view of the fundamental TE mode size of a standard single-mode fiber (SMF-28), the grating region was set 15-µm wide. A linear adiabatic taper was designed to be 300-µm long. The taper length may be shortened using a series of concatenated tapers^13^. Because the grating plays a pivotal role in the coupling of light from the fiber to the waveguide, its parameters were fastidiously derived. A cross-sectional view of the GAC is shown in Fig. 5b. The gratings were created by inscribing an array of grooves over a distance L_1_ according to a depth of d_g_, in a strip waveguide with a thickness of h_SiN_ = 500 nm. The fill factor of the grating is defined as the ratio of the uninscribed waveguide length (L_2_) to the total grating pitch (Λ). For an incident angle (θ) of 13°, the grating pitch is estimated using the Bragg’s condition to be approximately 1.15 µm for the fully inscribed condition with groove depth (d_g_) of 500 nm at ${\uplambda }_{{\text{c}}}$ = 1550 nm. FDTD simulations were executed to probe the dependence of the total coupling efficiency on the structural parameters. Figures 5c,d show the calculated coupling efficiencies with respect to the groove depth, pitch, and fill factor of the GAC. The GAC is observed to achieve the maximum efficiency when d_g_, Λ, and f are 500 nm, 1.14 µm, and 0.5, respectively. Noting the thicknesses of the upper cladding (h_UC_) and bottom cladding (h_BC_) affect the coupling efficiency^11,12^, simulations were carried out by varying h_UC_ and h_BC_, as shown in Fig. 5e,f, respectively. The maximum efficiency was obtained for h_UC_ = 3.7 µm and h_BC_ = 2.6 µm. The GAC was simulated to discover the throughput from the fiber to the waveguide using the specified parameters, as presented in Fig. 5g. The observed throughput was − 3.2 dB at λ = 1550 nm and remained beyond − 3.9 dB throughout the C-band.

**Figure 6:**
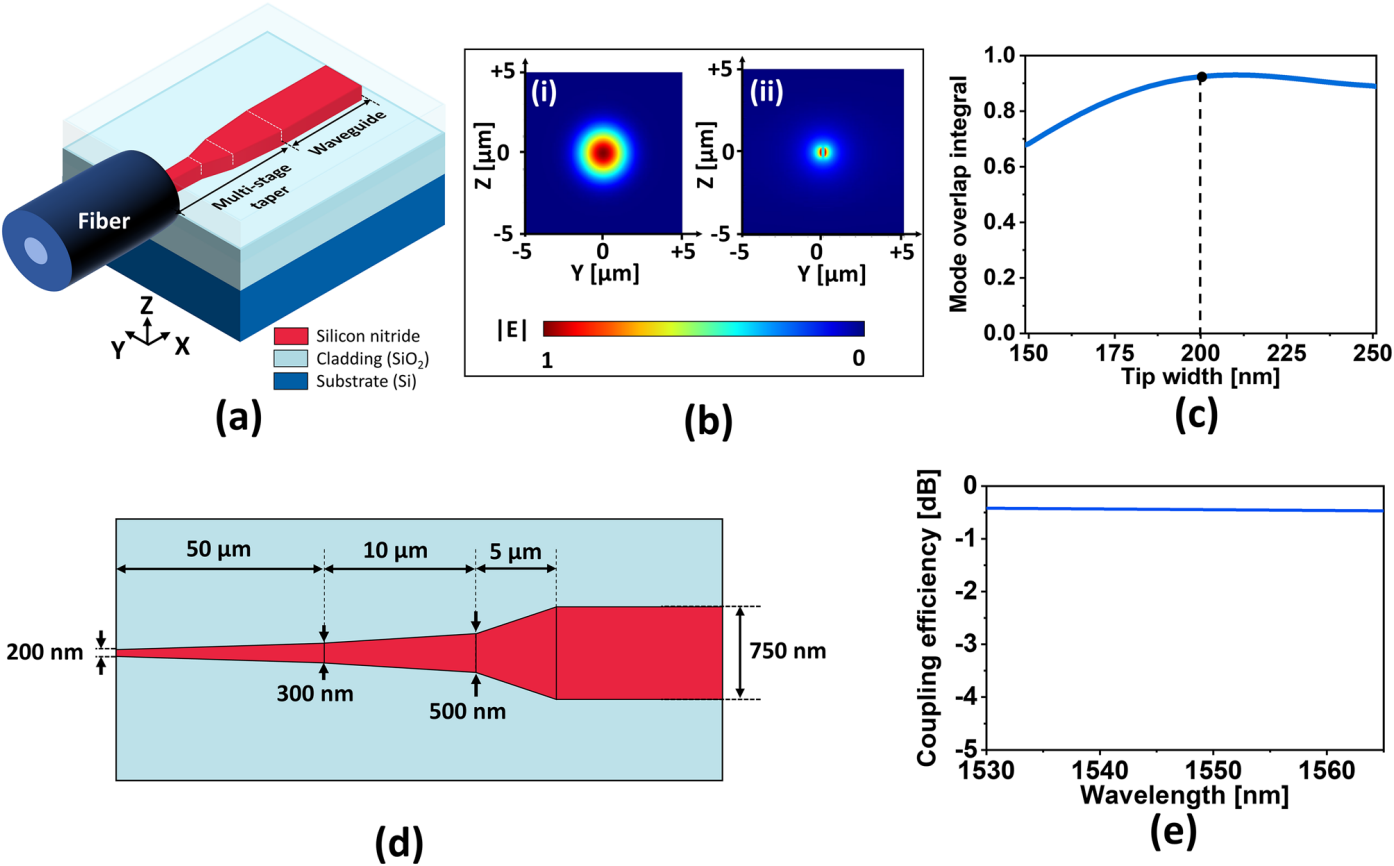
(**a**) Proposed end-fire coupler based on an SSC comprising a multi-stage taper. Calculated guided mode profiles of the (**b**)–(i) high-numerical-aperture fiber and (**b**)-(ii) the taper terminated with a 200-nm-wide tip. (**c**) Calculated mode overlaps between the fiber and taper tip in terms of the tip width. (**d**) Designed multi-stage taper consisting of three serially concatenated segments and (**e**) spectral coupling efficiency of the end-fire coupler based on the designed taper.

For end-fire coupling, an SSC was suggested to deliver light from the fiber to the waveguide. The SSC incorporated a multi-stage taper which could expand the guided mode profile of the waveguide to match that of the fiber. Here, the SSC was designed in line with an ultra-high numerical aperture fiber exhibiting a tiny mode field diameter of ~ 4 µm, whose splicing loss relating to a standard single mode (SMF28) fiber was known to be as small as 0.06 dB^24^. As shown in Fig. 6a, the SSC is based on a 500-nm thick SiN multi-stage taper linked to a 750-nm wide output waveguide. The thicknesses of the upper and the bottom cladding were chosen to be 2.6 µm and 3.7 µm, as per the GAC design. As for the taper, the mode overlap between the fundamental TE mode of the fiber and that of the tip of the taper was estimated. The TE mode field profiles of the fiber and the 200-nm wide tip waveguide are depicted in Fig. 6b-(i) and (ii), respectively. The mode overlap integral^25^ was scanned in terms of the taper tip width, as plotted in Fig. 6c, signifying that a tip width of 200 nm lead to an approximate maximum overlap of 0.94. Hence, a tip width of 200 nm was selected for the SSC. Rather than exploiting a single adiabatic taper, typically measuring several hundred micrometers in length, a shortened multi-stage taper drawing upon three serially concatenated taper segments was adopted^13^. As delineated in Fig. 6d, the proposed taper has a total length of 65 µm. Figure 6e shows the spectral response of the SSC coupling efficiency, signaling the SSC could deliver a coupling efficiency better than − 0.5 dB throughout the C-band. In order to enhance the tolerance in terms of dicing and polishing processes during practical device manufacturing, the SSC can take advantage of a short length of elongated tip waveguide, whose width is in the vicinity of 200 nm, as demonstrated in our previous work^13^.

## Analysis of the RFWC module

When the designed basic elements were prepared, the operation of the complete RFWC module was verified through rigorous simulations. First, a conjoined switchable directional coupler (C-SDC) was devised to facilitate switching at both the input and output sides. Second, the complex amplitude reflection and transmission coefficients (S-parameters) in relation to the constituting elements, inclusive of the GAC, SSC, and C-SDCs, were extracted to develop their models, which were assembled to establish the entire RFWC module.

### Design and performance analysis of the conjoined switchable directional coupler

**Figure 7:**
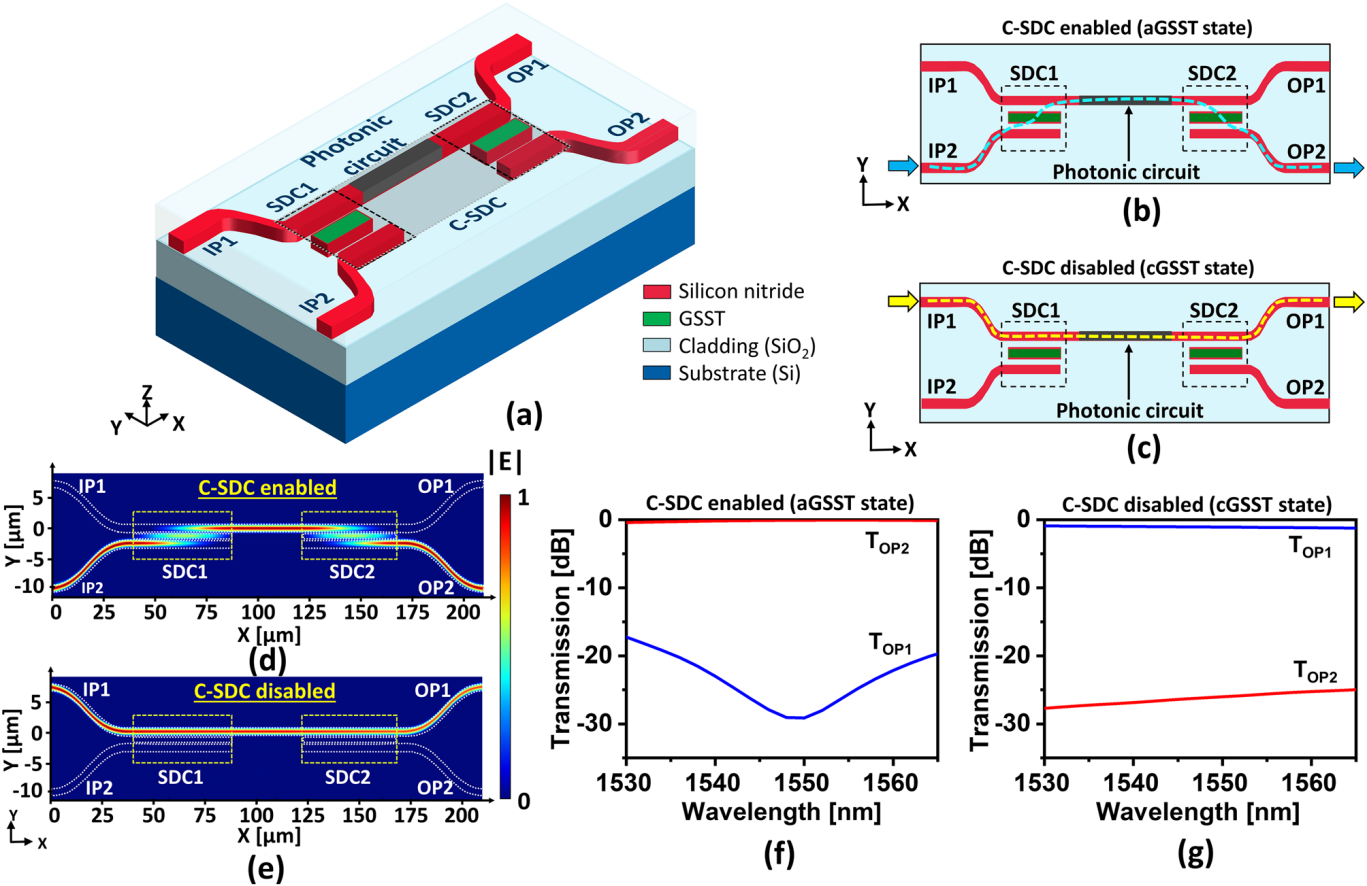
(**a**) Schematic of the proposed C-SDC where both SDC1 and SDC2 are linked to each other via a photonic circuit. The operation of the C-SDC (**b**) under aGSST state where input light from IP2 couples to the photonic circuit through SDC1 and couples back to OP2 through SDC2 and (**c**) under cGSST state where light from IP1 undergoes no cross coupling induced by SDC1 and SDC2, eventually exiting OP1. Optical coupling characteristics under (**d**) aGSST and (**e**) cGSST states. The transmission spectra of T_OP1_ and T_OP2_ as observed at the OP1 and OP2 ports for the cases of C-SDC overlaid with (**f**) aGSST and (**g**) cGSST, respectively.

As shown in Fig. 7a, the proposed C-SDC comprises two SDCs (SDC1 and SDC2) interfaced to either end of the photonic circuit enabling reconfigurable input/output coupling to and from the circuit. In this work, a straight waveguide acts as the photonic circuit. When the state of GSST belonging to both SDCs is aGSST, the C-SDC is enabled, thus allowing the input light from the IP2 port to couple via SDC1 into the circuit, which subsequently couples via SDC2 and exits through the OP2 port, as shown in Fig. 7b. Meanwhile, the C-SDC is disabled when both SDCs are in the cGSST state, which makes the input light from the IP1 port pass through the photonic circuit without experiencing cross coupling at either SDC1 or SDC2, and the light finally comes out of the OP1 port, as shown in Fig. 7c. Full simulations were conducted using a 3D FDTD scheme to examine the field propagation profiles and efficiencies. The field propagation profiles for the cases where the C-SDC was enabled and disabled are shown in Fig. 7d,e, respectively. The profiles corroborate the working mechanism of the C-SDC, as shown in Fig. 7b,c. The spectral response of the output power transmitted into ports OP1 and OP2 when the C-SDC is enabled is shown in Fig. 7f. The total IL of the C-SDC when passing through both SDCs is less than 0.6 dB throughout the C-band. The ER for the C-SDC-enabled case is as high as 26 dB at λ = 1550 nm and above 17 dB throughout the C-band operation. Similarly, when the C-SDS is disabled, the IL is less than 1.2 dB and the ER is above 24 dB, as shown in Fig. 7g. For further assessment of the proposed RFWC module, the performance parameters were exported in the form of S-parameters.

### Performance of the RFWC module

**Figure 8:**
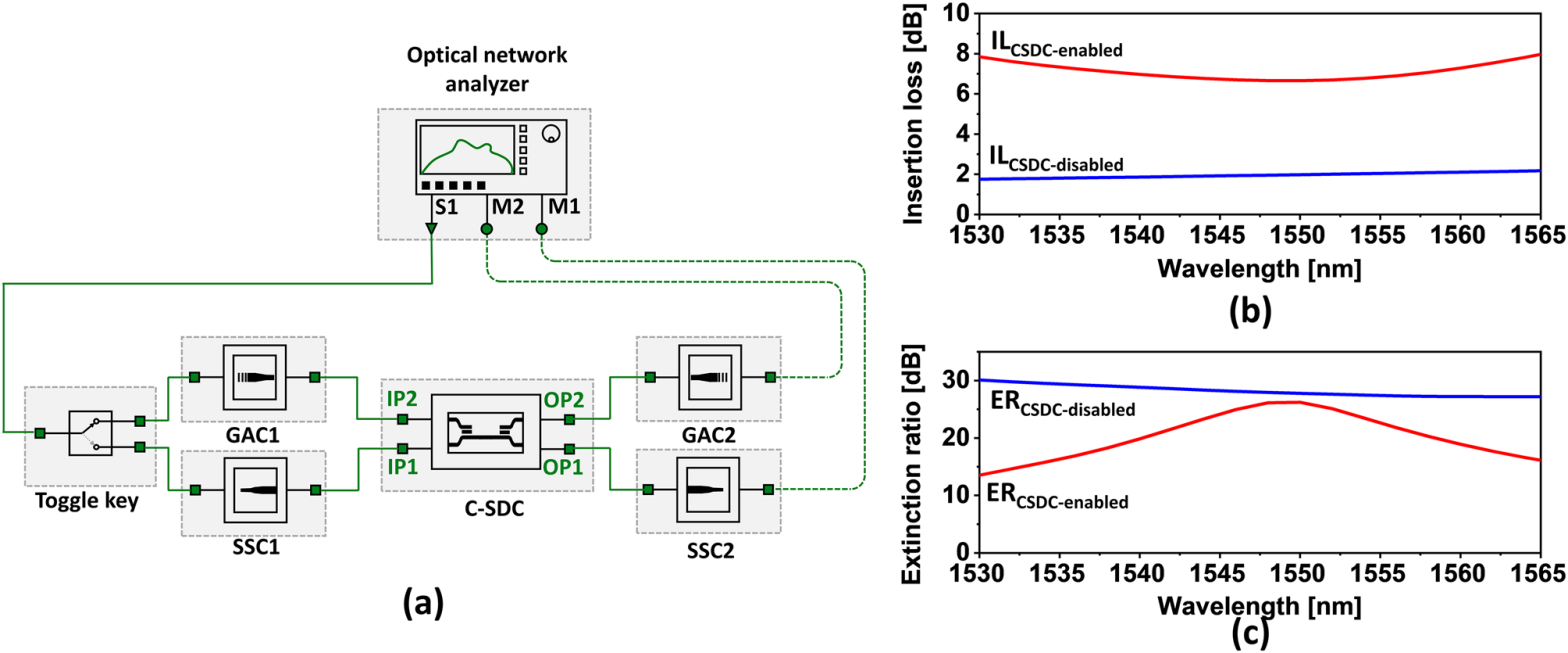
(**a**) Schematic layout portraying a complete RFWC module under simulation in which all functional blocks including the proposed GACs, SSCs and C-SDC are deployed. An optical network analyzer having a light source (S1) and two power monitor terminals (M1 and M2) is used to monitor the spectral transmission of the circuit. (**b**) Calculated IL spectra using the transmitted power recorded at the monitors M1 and M2 for both the cases of C-SDC enabled and disabled. (**c**) Calculated ERs of the circuit as observed at M1 and M2 of the analyzer, for the C-SDC-enabled case with the input/output through the GACs, and the case of disabled C-SDC with the input/output via the SSCs.

To validate the operation of the proposed RFWC module tapping into the grating/SSC couplers on top of the C-SDC, simulations were fulfilled using the Lumerical Interconnect tool, which has been widely used for full photonic circuit simulations. The S-parameter models of the constituting elements were derived and then interconnected to establish the proposed RFWC module, as delineated in Fig. 8a. Here an optical network analyzer is equipped with a light source (S1) to feed light into the circuit as well as two monitors (M1 and M2) for analyzing the output from the circuit. The toggle key is a representative unit that just conveys light from S1 to the input GAC1 or SSC1 during their simulations, not belonging to the proposed RFWC module. When the C-SDC is enabled, the toggle key allows the input light to impinge upon the C-SDC via GAC1. The input light then propagates through the photonic circuit in the C-SDC and eventually exits via GAC2. Meanwhile, for the case when the C-SDC is disabled, the input light is routed via SSC1 into the C-SDC and ultimately exits through SSC2. For both the cases when the C-SDC is enabled and disabled, the output light through both SSC2 and GAC2 are recorded to estimate the ER. It is noteworthy that the S-parameter models corresponding to aGSST and cGSST were applied to the cases where the C-SDC was enabled and disabled, respectively.

The operation of the RFWC module was examined in terms of IL and ER as well. The IL, alluding to the amount of power loss incurred by the coupling module, accounts for the loss caused by both the input and output couplers (either the GACs or SSCs) along with the IL of the C-SDC. The ER is relevant to the power that escaped to the output SSC2 when the C-SDC was enabled and to GAC2 when the C-SDC was disabled. The total IL and ER of the RFWC module were calculated according to ${\text{IL}}_{{\text{CSDC - enabled (CSDC - disabled)}}} {\text{ = 10 log}}_{{{10}}} {\text{ T}}_{{\text{GAC2(SSC2)}}}$ and ${\text{ER}}_{{\text{CSDC - enabled (CSDC - disabled)}}} {\text{ = 10 log}}_{{{10}}} {\text{ T}}_{{\text{GAC2(SSC2)}}} {\text{/ T}}_{{\text{SSC2(GAC2)}}}$, respectively. Here, T_GAC2_ and T_SSC2_ represent the output powers transmitted to the network analyzer through GAC2 and SSC2, respectively. The IL values resulting from the analysis of the RFWC module are shown in Fig. 8b. For the circuit in which the C-SDC is disabled and the inputs and outputs are through SSCs, the total IL_CSDC-disabled_ is less than 2.1 dB throughout the C-band. Similarly, when the C-SDC is enabled with the light input/output going through the GACs, the total IL_CSDC-enabled_ is below 7.8 dB throughout the wavelength regime of concern. It is mentioned the loss can be suppressed with the introduction of an apodised or multilayered GAC^10^. The practical implementation of the GACs based on a C-SDC might be undesirably affected by Fabry–Perot resonances between the grating region and the waveguide end in the coupling region. However, considering the proposed module can accommodate flexible schemes of GACs, it is expected that the resonance issue can be mitigated by taking advantage of focusing GACs, which are deemed to feature reduced back-reflection^12^.

The proposed coupling module was observed to exhibit a decent level of ER for both the C-SDC-enabled and disabled cases. Throughout the C-band, the ER_CSDC-enabled_ and ER_CSDC-disabled_ were consistently above 13 and 27 dB, respectively, as plotted in Fig. 8c. Although the C-SDC is chiefly responsible for the ER, the discrepancy in the ER between the RFWC module and C-SDC alone is attributed to the disparate coupling efficiencies of the GAC and SSC for the RFWC module simulation. It should be remarked that for the ER of the C-SDC alone, there are no input and output couplers, leading to no coupling loss; hence, the ER is exclusively affected by the SDCs. However, this case does not hold true for the RFWC module that involves a GAC and SSC inducing a certain amount of coupling loss. This discrepancy of ER between the C-SDC and RFWC module may be abated by embodying GACs that could provide a coupling efficiency tantamount to that of the SSC. Nonetheless, the ER and IL of the proposed RFWC are reasonable for practical applications toward photonic integrated circuits. Based on the ILs of the input and output SSC and GACs, the excess loss pertaining to the RFWC was below 0.4 and 1.2 dB when the C-SDC was enabled and disabled, respectively. Consequently, the proposed RFWC module is highly expected to be deployed in various photonic circuits, playing a vital role in concurrently accommodating both GACs and end-fire SSCs.

## Conclusion

A RFWC module that allows both the GAC and SSC to interrogate the same photonic circuit is proposed and demonstrated. The proposed module renders selective switching between the GAC and SSC input/outputs by leveraging the nonvolatile switching ability of the GSST-based directional coupler. Considering the state of GSST can be switched back and forth from amorphous to crystalline via low-temperature annealing under specific environmental conditions,^19^ the inputs and outputs can be switched back and forth from the GAC to the SSC and vice versa, not involving continuous power during the operation of the circuit. This flexibility in choice of the input and output coupler enables not only full-scale wafer-level testing via the GAC but also low-loss and broadband applications at the chip level when the chips are diced out of the wafer. In consideration of its minimal excess loss below 1.2 dB and decent ERs above 13 dB while operating under both states of GSST, it is claimed that the proposed RFWC module can be applied to practical photonic circuits without compromising the performance. Although the current demonstration is underpinned by a SiN platform in combination with a PCM of GSST, the proposed module can be readily tailored for other types of platforms and PCMs^14,16^. The RFWC module is slated to serve as a springboard to resolve the existing trade-off between full-scale wafer-level testing and broadband high-efficiency coupling at the chip level, paving avenues for pursuing various hybrid photonic circuits based on PCMs.

## Methods

We have used a finite-difference eigenmode (FDE) solver [Ansys Inc.] to reveal the effective refractive indices and field profiles of the directional coupler supermodes. The FDE solver calculates the mode field profiles, their effective refractive indices, group indices, and optical losses by solving the Maxwell’s equation across the meshed cross-sections of the waveguides. A three-dimensional eigenmode expansion (EME) solver from Ansys Inc. was adopted to determine the taper lengths and calculate the coupling efficiency of the end-fire coupler. The rest of the simulations were based on the three-dimensional full-vectorial FDTD solver. The S-parameters were exported for each of the elements, which were used to construct the complete module. We also resorted to a tool, Lumerical Interconnect, to conduct the full circuit simulations and assess the performance of the proposed reconfigurable coupler module. For the simulations, the refractive indices of the SiN and SiO_2_ were assumed to be 2.0 and 1.44 at a wavelength around 1550 nm, respectively. The complex refractive indices of both the amorphous and crystalline GSST were derived from the dispersion properties based on the experimental work by Zhang et al.^21^.

## Data Availability

The datasets generated and/or analyzed during the current study are available from the corresponding author on reasonable request.
